# Odor Detection Using an E-Nose With a Reduced Sensor Array

**DOI:** 10.3390/s20123542

**Published:** 2020-06-23

**Authors:** Piotr Borowik, Leszek Adamowicz, Rafał Tarakowski, Krzysztof Siwek, Tomasz Grzywacz

**Affiliations:** 1 Faculty of Physics, Warsaw University of Technology, ul. Koszykowa 75, 00-662 Warszawa, Poland; pborow@poczta.onet.pl (P.B.); Rafal.Tarakowski@pw.edu.pl (R.T.); 2 Faculty of Electrical Engineering, Institute of Theory of Electrical Engineering, Measurement and Information Systems, Warsaw University of Technology, ul. Koszykowa 75, 00-662 Warszawa, Poland; Krzysztof.Siwek@ee.pw.edu.pl (K.S.); Tomasz.Grzywacz@ee.pw.edu.pl (T.G.)

**Keywords:** electronic nose, features selection, odor classification, sensor array reduction, wine spoilage

## Abstract

Recent advances in the field of electronic noses (e-noses) have led to new developments in both sensors and feature extraction as well as data processing techniques, providing an increased amount of information. Therefore, feature selection has become essential in the development of e-nose applications. Sophisticated computation techniques can be applied for solving the old problem of sensor number optimization and feature selections. In this way, one can find an optimal application-specific sensor array and reduce the potential cost associated with designing new e-nose devices. In this paper, we examine a procedure to extract and select modeling features for optimal e-nose performance. The usefulness of this approach is demonstrated in detail. We calculated the model’s performance using cross-validation with the standard leave-one-group-out and group shuffle validation methods. Our analysis of wine spoilage data from the sensor array shows when a transient sensor response is considered, both from gas adsorption and desorption phases, it is possible to obtain a reasonable level of odor detection even with data coming from a single sensor. This requires adequate extraction of modeling features and then selection of features used in the final model.

## 1. Introduction

Detection and analysis of smells among specified applications can be assessed by many analytical techniques. Classical methods of chemical analysis, such as gas and liquid chromatography, mass spectrometry, nuclear magnetic resonance, and spectrophotometry, are highly reliable, although they are expensive, time-consuming, and unsuitable for on-site monitoring. Over the last two decades, one can observe a rapid expansion in the development of artificial organoleptic systems [1], so called electronic noses (e-noses). Various sensing methods based on thermal, optical, gravimetric, and electrochemical techniques have been developed since the introduction of the e-nose concept. Particularly promising is surface plasmon resonance imaging [2] and its successful application for gas-phase detection of volatile organic compounds [3].

These new electronic instrumentations are capable of imitating the remarkable abilities of the human nose, and they have proved their feasibility and effectiveness in odor recognition, environmental monitoring [4], medical diagnosis [5], as well as food quality monitoring [6,7,8,9]. Numerous important papers regarding wine odor recognition by electronic nose have also appeared [9,10,11,12,13,14,15,16,17,18]. In the present paper, we will focus on data concerning wine quality [19,20].

An e-nose is a rapid, noninvasive, and intelligent on-line instrument. It comprises an array of carefully chosen sensors and an appropriate pattern recognition system capable of identifying particular smells. There are distinct types of e-noses in different application fields based on commercially available sensors. Nevertheless, numerous challenging questions arise related to performance enhancement including improvement of classification rate, rapidity of on-line detection, and recognition and prediction accuracy [21]. Each system has its own advantages and disadvantages in improving the performance of e-noses. However, there exists another possibility for increasing the sensitivity, selectivity, response and recovery time, and detection and operating range, which is instrumentation independent. The issue has appeared in the context of false classifications due to sensor signal drift, temperature, humidity, and other factors [22]. The sensitivity and signal-to-noise ratio (S/N) are fundamental features of individual sensors used in e-noses. They are determined by the current state of technology. On the other hand, the optimal number and types of sensors in a given detection system are still open to debate. In practice, the answers depend mainly on the experience and intuition of researchers involved in the implementation, as no general rule exists. Verification of various sensor combinations leading to the best classification performance is employed. In general, it is best to use as few variables as possible to develop a model, as this results in a higher ratio of data points to variables [22]. In this paper, we employed a simple procedure that assures adequate extraction and selection of modeling features and determines the optimal number of sensors.

An e-nose is both a sensing and data analysis system designed to discriminate between different odors. Recent advances in the field of e-noses have led to new developments in both sensors and feature extraction as well as data processing techniques. Consequently, users of a multiple sensor instrument are provided with an increased amount of information. Therefore, for the development of e-nose applications it is essential to deeply examine problems related feature selection, by removing redundant sensors that are possibly adding noise into the system, instead of improving discrimination. Evolutionary computation techniques can be applied to optimize sensor selection, feature selection, and classification stages [23]. In this way, one can find an optimal subset of sensors for a particular application while choosing sensing devices from a larger database of sensors. These techniques help to create smaller application-specific sensor arrays and help to reduce the potential cost associated with new sensor developments to solve complex olfactory problems. Optimization of electronic nose sensor arrays and the choice of the appropriate subset of sensors have been reported by many researchers [24,25,26,27,28,29,30].

We would like to consider the particular application of using a reduced sensor array in odor recognition. It is known that replacement of faulty sensors requires device recalibration [31], which is often associated with new data collection and model retraining. There is some research on mitigating this problem [32] by using dedicated algorithms with a smaller number of sensors. One can ask how far such a procedure can be continued and how small an array of sensors could be to maintain correct classification of odors by an electronic nose. Some authors [33,34,35,36,37] reported the possibility to recognize odors using data collected by only one sensor. They proposed experimental set-ups, which exploited transient responses to the measured gas exposure in both adsorption and desorption phases and also explored larger regions of the sensor response characteristics, taking advantage of temperature modulation [36,37] or disturbances in sensor exposure to gas conditions [35]. In our paper, we explore the feasibility of odor recognition using single-sensor data coming from a simple experimental setup.

This paper is organized as follows. In Section 2 we describe the electronic nose device constructed by Rodrigues Gamboa [19] and co-workers as well as measurements they performed. In the following Section 3, modeling techniques implemented in our method are introduced. Then, the results of our modeling are discussed in Section 4. We summarize our findings in Section 5.

## 2. Odor Measurements by Electronic Nose

In this paper, we used publicly available data sets [20] of wine quality measured via e-nose. Results focused on rapid detection of wine spoilage have been published by Rodriguez Gamboa et al. [19]. The authors demonstrated that the support vector machine model is able to correctly classify four types of studied odors with an accuracy up to 97%. The published results of another machine learning model, which applied a multi-layer perceptron neural network, demonstrated the ability to rapidly classify odors using only initial points of e-nose measurements a few seconds after sensor were exposed to the odor.

In the present paper, we focus on different feature selection issues used in modeling, especially when data from a reduced number of sensors are used. Even though details of electronic nose construction and measurements have already been published by Rodriguez Gamboa and co-workers [19,20], for the reader’s convenience we would like to present here a short summary.

### 2.1. Electronic Nose

The e-nose developed at Universidade Federal Rural de Pernambuco [20] consists of six commercially available metal-oxide gas sensors produced by Hanwei Sensors (www.hwsensor.com). Two sensors of each type were used in the presented construction: (i) MQ-3, highly sensitive to alcohol, with low sensitivity to benzine (sensors 1 and 4); (ii) MQ-4, highly sensitive to CH4 and natural gas (sensors 2 and 5); and (iii) MQ-6, highly sensitive to LPG, iso-butane, and propane (sensors 3 and 6). Metal-oxide sensor responses vary between individual devices, which can be observed by the differences in resistance values of measured gases as well as in differences between transient sensor characteristics. Such differences can be exploited in e-nose construction when several sensors of the same series are used for odor recognition tasks.

The following data collection procedure was applied. At the beginning of measurements, a small amount (1 mL) of wine sample was put in a concentration chamber, and the volatile compounds were collected for 30 s. The first stage of measurement, lasting 10 s, was used to collect the baseline sensor response when the e-nose was exposed to pure air. Then, prepared gas with the sample odor was pumped into the sensor chamber for 80 s to measure the response during the adsorption phase. After that, pure air was again pumped to the sensor chamber, and the response during the gas desorption phase was also collected for 90 s. The sampling rate was set to 18.5 Hz, and transient sensor resistance values were collected. After that, the set-up was exposed to pure air for 600 s to purge volatile residues and relax the sensors.

**Figure 1:**
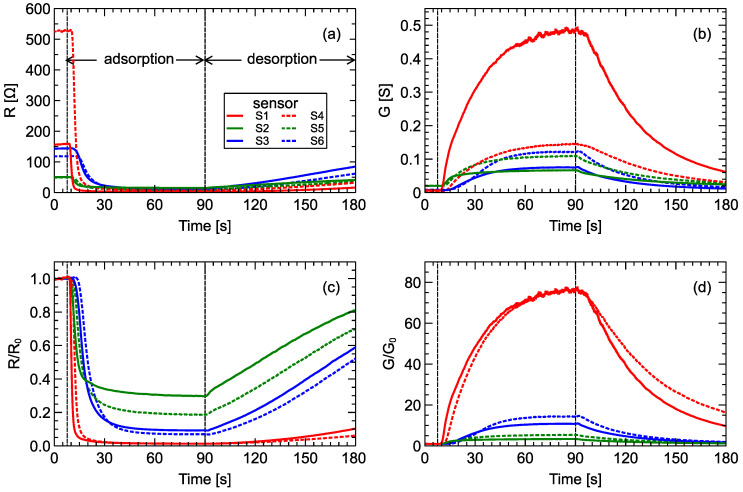
Typical sensor array response: (**a**) sensor resistance and (**b**) sensor conductance. Sensor responses standardized to the baseline measurements (**c**) $R/R_{0}$ and (**d**) $G/G_{0}$. Vertical lines indicate various phases of the measurement process: baseline collection, gas adsorption, gas desorption. To not overload charts, assignment of all lines to the sensor numbers is indicated only in chart (**a**); line styles are consistent in all charts. Data measured by sensor pairs of the same type are plotted using the same color with different line styles. Measurements of average-quality wine (AQ_Wine02_B04_R01) were used in this figure.

In Figure 1 we present the typical response of all sensors in the sensor array. First, in Figure 1a the resistances of individual sensors as a function of time are displayed. The same information is contained in sensor conductance curves Figure 1b, but such transformation may be useful to reveal more patterns in the data, so these values were also used in data analysis. As expressed by metal-oxide sensors, meaningful measurements include responses relative to pure air, so they are presented in Figure 1c,d. As mentioned above, sensor discreetness can be easily noticed. Even if pairs of sensors from the same series are used, the difference in response is distinguishable when exposed to the same gas conditions.

### 2.2. Measurement of Wine Odor

**Figure 2:**
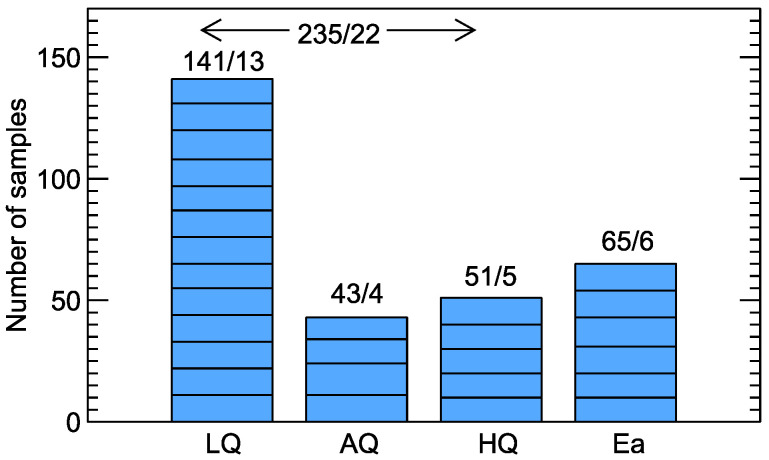
Number of examined samples/number of bottles for various studied wines categories. LQ—low quality, AQ—average quality, HQ—high quality, and Ea—diluted ethanol. Number of samples from each bottle is represented by bar segments.

Rodriguez Gamboa and co-workers [19] measured wine odors at various stages of spoilage. The experiments were performed with 22 bottles of commercially available wines, of different varieties and vintages, elaborated in four wineries of the São Francisco valley (Pernambuco, Brazil). Of these bottles, 13 were randomly selected and left open for 6 months, which gave the population of low-quality wines (LQ). Another four randomly selected bottles were left open for 2 weeks before measurement, and they were labeled as average-quality wines (AQ). The remaining five bottles were considered high-quality wines (HQ). Besides wine samples, six different concentrations of ethanol diluted in distilled water were also used, which may be considered as six additionally measured bottles. Between 10 and 11 samples from each bottle of wine and between 10 and 12 samples from each bottle of diluted ethanol were taken for measurements. The number of measured samples in each category is graphically presented in Figure 2.

## 3. Classification Modeling

Odor recognition using electronic noses, from the data processing point of view, is a classification task using machine learning models. In this section, we describe various elements of our approach including extraction of features used for modeling (Section 3.1), validation of classification model performance (Section 3.2), employed modeling technique (Section 3.3), and feature selection by wrapper and filter methods (Section 3.4).

For the calculations presented in this paper we developed programs in Python 3.7 language using the scikit-learn module [38] .

### 3.1. Extraction of Modeling Features

One series of gas measurements using the electronic nose consisted of hundreds or thousands of individual sensor response points over time. In Figure 1, we present the entirety of sensor measurements for a single sample, which consisted of 3300 measurement points for each sensor in addition to the baseline measurement. Summing up, for all sensors, there was a space of 19,000 dimensions. This is impractical for model building, so a smaller number of features characterizing response curves was extracted.

The most basic feature that can be used for classification is the final steady-state value of the response curve after sensors are exposed to the studied odor: $G_{max}/G_{0}$ in the case of conductance and $R_{min}/R_{0}$ in the case of resistance. However, this means that in such a case only one value in the whole response curve is extracted, and information of the transient response is not used in modeling. Other features [39] include basic statistics calculated from the response characteristics such as the average value (which, in the case of keeping the same data collection frequency and time span for all measurements, is equivalent to the integral or area below the response curve), standard deviation, skewness, and kurtosis. In some research, sensor response values in selected moments of time are used. They are usually evaluated after smoothing the curve in order to remove measurement noise. In a similar spirit, capturing data in a moving window function [40,41] is applied. In several studies [19,42,43] the exponential moving average (emaα) of the response curve is used, and its maximum/minimum values for several smoothing parameters α are extracted as modeling features. Related to these are features used in other works such as extreme values of the response curve derivative [44,45,46]. The response curve can also be approximated by analytical functions such as polynomial, sigmoid, or exponential, and the fitted parameters can be used as the modeling features [44,45]. Additionally, characteristic times, such as the time to reach maximum/minimum of the curve derivative or time to reach, for example, 10%, 25%, or 50% of the sensor response range, can be used as modeling features. Yan et al. [47] reviewed applications of various feature extraction methods in the odor detection domain using an electronic nose. All features extracted from the responses curves, which are used in the present work, are listed in the Appendix A.

Frequently, another approach is used to reduce the dimensionality of the modeling problem. The measurement data or extracted features are projected to a lower-dimensionality space as linear or nonlinear transformations of the original data. After that, only a few most relevant, transformed variables, containing most of the information without noise, are used for model training. Probably the most often used method for such a task is Principal Components Analysis (PCA), but other methods can also be used.

**Figure 3:**
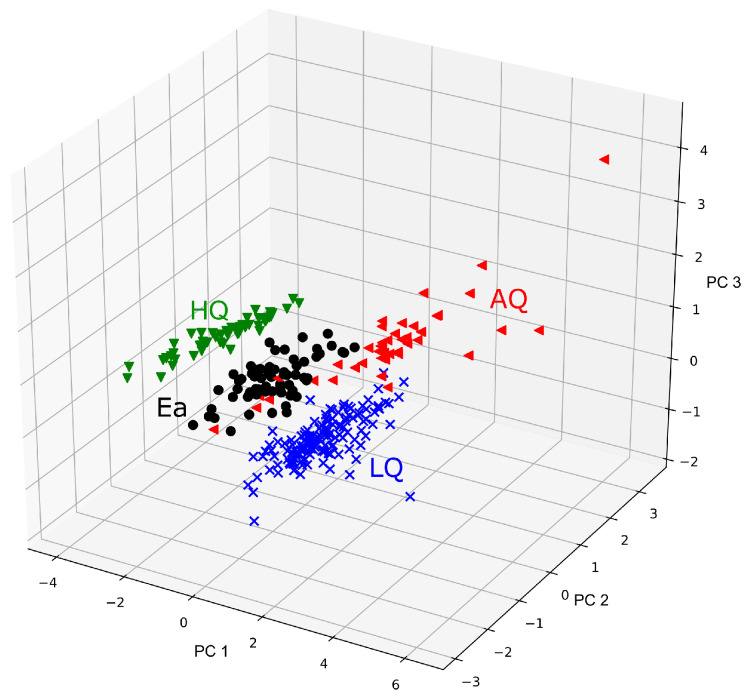
Example of transformed feature characteristics extracted from the sensors by Principal Components Analysis (PCA). Features from all sensors are considered, and the best five features selected by the forward selection method are used as input to PCA. The four studied wine categories are marked by colors: LQ–low quality, AQ—average quality, HQ—high quality, and Ea—diluted ethanol.

It should be noted that this type of dimensionality reduction is often used for other purposes. Even if the model is trained on the original features, the transformed feature space by PCA can be used to visualize data patterns and clusters that appear in various categories. In this spirit, in Figure 3 we demonstrate that the selected features were able to discriminate the studied categories of wine odors. As one can notice, the high-quality and low-quality wines were clearly separated; however, there was noticeable overlap between average-quality wines and ethanol sample measurements.

### 3.2. Model Validation

An important part of the machine learning modeling process is validating the model’s performance, which should be done on a dataset independent from the dataset used for model training. As one might notice in the description of examined odors in Section 2.2, there can be correlation between the studied wine samples because they come from the same bottle. Rodriguez Gamboa et al. [19] proposed to use a cross-validation procedure with the “leave-one-group-out” scheme. Measurements performed on samples coming from one bottle were kept apart for testing the model performance, and the rest of the data were used for model training. This procedure was performed in a loop over all bottles, and the performance results were averaged.

In our modeling, we opted for another validation scheme and implemented the “group shuffle split” method. In this case, the available dataset is split into training and validation sets in a 75/25% proportion, with a restriction that all samples coming from a particular bottle should be in either training or validation sets. This was based on random selection; thus, in order to obtain more reliable metrics of the model performance, the procedure was repeated 100 times and the results were averaged. We verified that the results were not affected by increasing the number of repetitions. We also verified various proportions of the training/validation split, and the chosen value gave optimal results in terms of the spread of individual model performance.

The reason to chose different approaches is as follows: The model’s performance calculated by the leave-one-group-out method was about 2–3 percent points higher than the one obtained by the group shuffle split approach. However, the standard deviation of the performance metrics by the group shuffle split approach was significantly lower. The difference between results of the considered cross-validation methods was smaller than the standard deviation. We argue that the chosen approach gives more reliable results.

### 3.3. Modeling Technique

Various machine learning techniques can be used [48] for the task of odor recognition using an electronic nose. In the studies from Reference [19] we refer to, classical methods of support vector machine and multilayer perceptron deep neural network were applied. Results in this paper were obtained using a multinomial logistic regression model. We performed a few tests using other modeling techniques such as support vector machine, decision tree, and k-nearest neighbors. The model performances were not superior to those obtained by logistic regression. However, for the other methods we did not perform so many tests as presented in this paper.

### 3.4. Feature Selection

The features used for the model are correlated, and the information contained in a set is redundant or often irrelevant to the considered classification task. If the subset of features is discriminated well, the studied classification categories a model trained on such subset usually exhibit better performances compared to the model in which all prepared features are used.

There are two main groups [49] of methods commonly used to select the most relevant features: wrapper and filter methods. Wrapper methods rely on the machine learning classification model in order to estimate the predictive performance of a subset of features. Several feature subsets are used for model training, then their performances are compared and used to select the best representation of the modeling features. A variant of the wrapper method is to select features by the model training algorithm itself, as it is, for example, in the case of decision tree [50]. Filter methods are independent of any predictive modeling algorithms. They rely on data characteristics to assess feature importance. These methods are typically more computationally efficient, but on the other hand, the selection of features by wrapper methods usually leads to better performance [24,25] of odor recognition and classification by electronic noses.

The main results in this paper were obtained with the wrapper-based approach. In many works, the recursive backward selection method has been applied [19,51], in which, first, a model based on all *N* features is created. Second, models based on N−1 features are also trained, and their performances are compared with the aim to choose which variable is the least important and upon removal will lead to the best model among all models based on N−1 features. Such a procedure is repeated recursively.

In our work, we implemented the recursive forward selection scheme. At the beginning, we compared models built just on a single feature, and the best of them was selected. In the next step, we compared models built on all combinations of two features in which the best feature from the first step was included and the best model determined which feature should be selected. This procedure was repeated recursively. We opted for this method as it is computationally less expensive compared to the backward selection method. The total set of modeling features consisted of several hundred items, and the expected number of features, for which models exhibited the best performance, was in the range of a dozen or so. Thus, the number of trained and compared models in the forward selection method was much smaller. As a metric for model comparison we used accuracy statistics (number of properly classified records/all number of records) calculated on the validation dataset. It should be mentioned that the variant of the forward feature selection method, specifically designed for the support vector machine model, has been used for electronic nose data by Gualdrón et al. [52].

We should keep in mind that both the backward selection and forward selection methods do not guarantee that the optimal subset of modeling features, leading to the best possible model performance, is found. The fulfillment of such a hard task would require comparisons of all possible subsets of the whole set of features (exhaustive search), which, even for moderate values of *N*, is computationally prohibitive and requires training and comparison of $2^{N}$ performance models. Some authors propose to use genetic algorithms to select the features subset [23,24,53], but this approach is beyond the scope of the present research.

We also implemented feature selection methods according to the filter approach [49]. This procedure consists of two steps: first, feature importance is ranked according to selected evaluation criteria, and second, the desired number of highly ranked features is chosen to train the model. In our research we used univariate methods, which means that each feature is ranked individually regardless of other features. We disregarded multivariate methods, in which several features are evaluated together in a batch. In our opinion, their potential advantage in the particular case of electronic nose data is not as important as in the case of big data problems. The size of the usually available training datasets is in the range of hundreds or thousands. In such a case, the time to train the model required for wrapper methods, which usually provides a better selection of features in terms of model performance, is acceptable on modern computer hardware.In our work, the Mutual Information, Fisher Score, and RelfiefF methods [49] were used for comparisons with recursive forward selection method described above.

We would like to emphasize works of other authors in which the application of filter feature selection methods relevant to electronic nose data have been reported. Mutual Information was applied, for example, by Wang and co-workers [54,55]. Nowotny et al. [56], Yin et al. [57], and Sun et al. [30] used multivariate Wilks’ Λ statistics and Mahalonobis distance to optimize the sensor array.

As a final remark in the description of the modeling procedure, we would like to add that all the above-described calculations were repeated five times, with different random number generator seeds, and the final results were averaged. It should be explained that this is not equivalent to the group shuffling cross-validation method described above, which works “inside” the recursive forward selection algorithm. Our averaging was performed “outside” of the cross-validation algorithm. As many modeling features are strongly correlated and there is some randomness in each repetition of the forward selection, different sets of features can be chosen, even if the overall model performance is similar.

## 4. Results

Model performance was studied using the standard leave-one-group-out cross-validation method. We obtained an average model accuracy equal to 96.6%, but for such a validation scheme, the standard deviation of the model was equal to approximately 11% (percent points). When the chosen validation scheme was group shuffle, with the proportion of training and validation datasets of 75/25% and 100 repetitions, the estimated model performance was smaller, equal to 94.3%, with a standard deviation of 7.5%. We performed both these calculations on all extracted features without selecting the best ones. In our opinion, the group shuffle method more reliably estimated the model’s performance, and the remaining results were obtained in this way.

**Figure 4:**
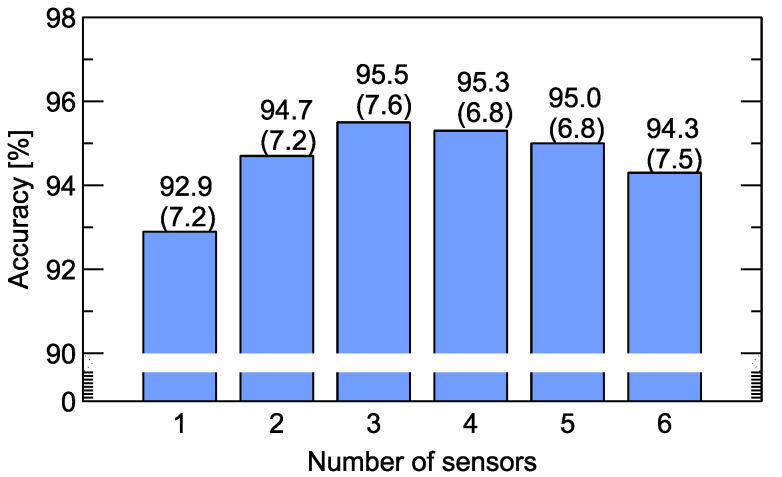
Accuracy of odor classification for various numbers of sensors from which data are used for model training. All training variables for these sensors listed in Appendix A are used. Above the bars, the average model accuracy and standard deviation (in brackets) are indicated.

In Figure 4 we present results when only part of the data is used for training the classification model, namely the data collected by a smaller number of sensors. We performed calculations for all combinations of sensors. Then, the optimal value for each number of sensors was selected. In these calculations all types of modeling features were used without feature selection. The results in this figure indicate that the optimal sensor matrix consisted of three sensors. However, it has to be mentioned that the difference between the choice of three and four sensors was very small (0.2%). Comparing this to the total number of measured samples equal to 300, this means that it was below the misclassification for just the measurement of one sample. We performed the same modeling several times, using different seeds of the random number generator, and in some of them the optimal choice of the sensor matrix consisted of four sensors.

**Figure 5:**
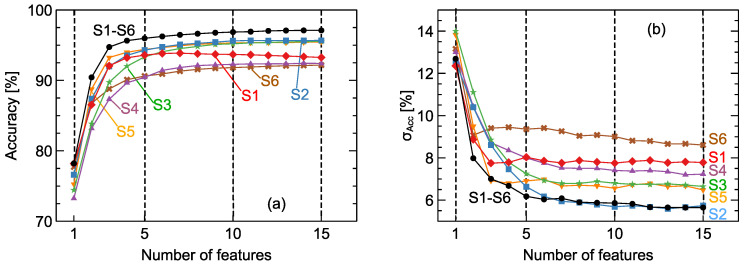
Average model accuracy (**a**) and standard deviation $\sigma_{Acc}$ (**b**) versus the number of features selected by the recursive forward selection method. Comparisons of models built on data from all sensors (S1–S6) with models trained on data from a single sensor (S1, …, S6). Lines are drawn as a guide for the eye.

In Figure 5a, we present the comparison of model accuracy obtained with various numbers of features chosen by the recursive forward selection method. First of all, the case when all types of features listed in the Appendix A are available for selection is presented. We can notice that the accuracy of the model built on only five features reached a value of 96.0%. Further increasing the number of features can still improve the model performance: when 10 features were used it reached 96.9%, and for 15 features it was 97.1%. Importantly, the performance obtained on reduced numbers of features, even when only five were used, was better than the best model accuracy obtained on the whole set of features as we presented in Figure 4. For machine learning models we would rather prefer models trained on a smaller number of features, as they will be more stable and less prone to overfitting. For such a reason, we insist that no more than 10 features is the optimal choice in this case. In Figure 5b, we also present the standard deviation of model accuracy obtained by the cross-validation procedure. What is more, the results demonstrated that the choice of more than 10 modeling features did not improve model stability.

Looking at the results presented in Figure 4, we can observe that the employment of a smaller number of features not only led to better model performance in terms of accuracy, but it also led to a significant improvement in model stability, with a cross-validation standard deviation of 5.6%. In turn, this means we can expect such a model will exhibit better performance on new, unseen data.

In the final remark of Section 3.4, we noted that we repeated the recursive forward selection procedure five times using different seeds of the random number generator and then averaged the results. Due to randomness, in each repetition the selected list of best features can be different. In our tests, the model with best choice of features gave an accuracy of 98.4% with a cross-validation standard deviation of 3.4%. This result is better than that reported in [19]. However, to present other results we used a more conservative approach when averaging the cross-validation estimations of model performance. The list of features selected by the modeling algorithm is presented in Appendix B.

**Figure 6:**
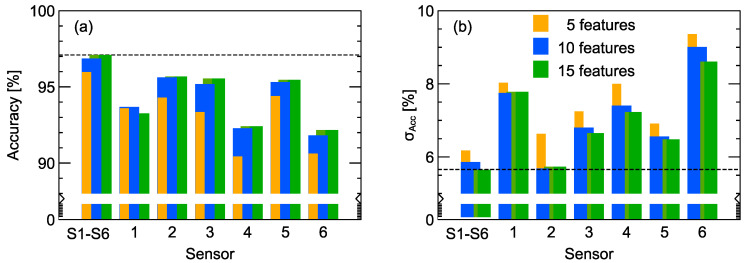
Average accuracy (**a**) and standard deviation $\sigma_{Acc}$ (**b**) of models trained on features extracted from all six sensor signals and from each individual sensor (1 to 6), respectively. Individual bars from left to right in each group display values obtained for 5, 10, and 15 features, respectively. A horizontal line is drawn to guide the eye and indicates the best performing model obtained with data extracted from responses of all sensors.

In Figure 5 we also present the performance of models trained on subsets of available features when, for the recursive forward selection method, only features extracted from a single sensor were used. Parts of these data are present in another form in Figure 6, where the average model performance and its standard deviation are plotted for the cases when 5, 10, and 15 features are used. We can notice that for the case of data extracted from just one sensor, namely, the 2nd, 3rd, and 5th sensors, the model accuracy was about 95.5%, which is very close to the performance of models trained on features extracted from all sensors. In Figure 6b we can also observe that, for the models trained on data extracted from only the 2nd sensor, the standard deviation, which can be interpreted as a kind of model stability, was comparable to the one obtained for the model trained on all data coming from all sensors.

Another instructive observation from that figure is the influence of individual sensor characteristics. As described in Section 2.1, there were three pairs of sensors of the same type. Sensors 2 and 5 were MQ-4 sensors designed to be highly sensible to CH${}_{4}$ and natural gas. The features extracted from both gave some of the best results. Yet, the difference between these sensors was distinguishable. When data from only the 2nd sensor were used, the models exhibited a significantly better stability than models trained on the data obtained from other sensors. On the contrary, sensors 3 and 6 were also of the same type, but as one can notice, the models trained on features extracted only from the 3rd sensor had good performances, but for the 6th sensor this was not the case.

**Figure 7:**
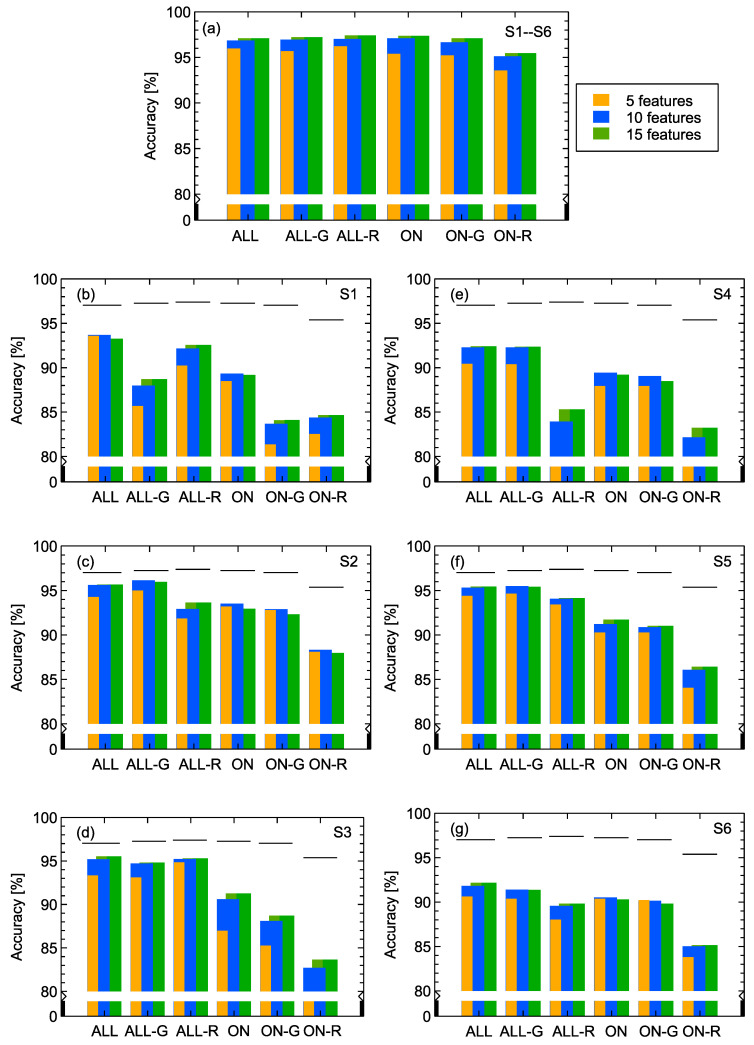
Accuracy of models trained on various subsets of features available for the recursive forward selection method: ALL—all prepared types of features, ALL-G—only features extracted from the conductance, ALL-R—only features extracted from the resistance, and ON, ON-G, ON-R—only features extracted from the gas adsorption part of the response. (**a**) Data from all sensors are used, (**b**–**g**) data from individual sensors are used for model training; sensors are indicated in sub-figures. Individual bars from left to right in each group display values obtained for 5, 10, and 15 features, respectively. Ticks above bars in figures (**b**–**g**) indicate the level of model accuracy obtained with all sensor data, as presented in figure (**a**). Charts (**b**–**g**) are ordered such that data from the same type of sensor are presented by row.

In Figure 7 we present results with the aim to address two other issues concerning data transformation and selection. As was already mentioned, we extracted modeling features from sensor response characteristics. The original measurements provide information in terms of sensor resistance *R*, but in our work we extracted features not only from the resistance but also using sensor conductance *G*, which is just the inverse of *R*. This means in the prepared set of features there are always pairs that are strongly correlated by this transformation. Of course, we can expect that the implemented features selection method will choose the most appropriate ones. Our intention was to verify if there was any important difference between models trained on features extracted from only *R*, only *G*, and both *R* and *G*.

The second issue we wanted to address is the possibility to use only part of the measurement characteristics from which the modeling features were extracted, precisely only the gas adsorption part of the measurement.

One can observe in Figure 7a that when data from all sensors were used for model training, the model accuracy did not depend on the choice of features extracted from G,R, or both. Additionally, only the adsorption part of the characteristics was sufficient; however, in such a case to achieve good model performance, it is required to use features extracted from sensors conductance.

When we examine in Figure 7b–g, which is related to models trained on data extracted from a single sensor response, we can notice that the best performing models were trained on the whole response curve (adsorption + desorption). In addition, transformation of the sensor resistance, in order to use sensor conductance to extract the features, leads to improved model performance, which can be especially noticed in sub-figures related to the 2nd and 5th sensors. These observations confirm that information contained in the desorption curve is valuable and can improve the odor recognition capability of an electronic nose. Additionally, using nonlinear transformations of both *R* and *G* related features can be helpful.

Goodner et al. [22] investigated the dependency of the classification model on the number of used sensors signals and especially on the relation between them and number of observations used to train the model. They performed numerical experiments when artificial noise had been added to the signal and came to the conclusion that, in general, a 1:6 ratio or higher is desirable. As one can notice, in the case when variable selection was used in our calculations, such a relation is fulfilled. However, it may be interesting to notice that even if all created features were used to train the classification model, the classification accuracy was still acceptable. That may seem to contradict the mentioned guidelines. This can be explained by the fact that, in the case of modeling features that we used, their values as well as noise that they included were strongly correlated. The best example was the correlation between features calculated as an integral of the response curve (area under the curve) with adsorption and desorption integral parts of the curve. Notably, the former is just a sum of the two latter. In the analysis presented in [22], the noise contained in all features was independent.

**Figure 8:**
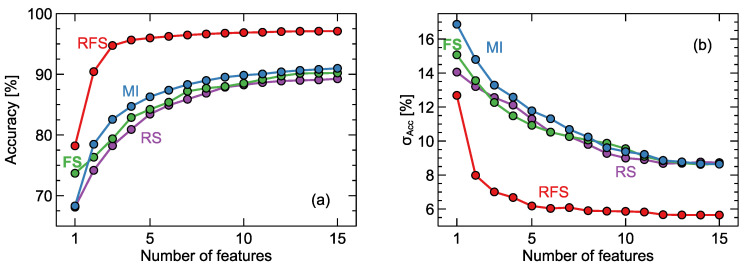
Average accuracy of models versus number of features used for training. Comparison of the recursive forward selection (RFS) method with filter selection methods: Mutual Information (MI), Fisher Score (FS), and ReliefF Score (RS). (**a**) average accuracy, (**b**) standard deviation of accuracy $\sigma_{Acc}$. Lines are drawn as a guide for the eye.

Finally, the results presented in Figure 8 demonstrate the advantage of choosing the wrapper feature selection method (recursive forward selection) over filter selection. We present here the model performance only for the case when data from all sensors were used. We performed calculations also for the cases of models built on individual sensor responses, and the characteristics of the results were similar.

## 5. Conclusions

In this paper, different issues concerning features selection associated with electronic nose applications have been considered. Particular attention has been devoted to data reduction by eliminating excessive numbers of sensors employed in the electronic instrumentation of a given e-nose. From the data processing point of view, odor recognition by using an e-nose is a classification task assisted by machine learning models. A number of approaches have been used for examining extraction of features, validation of model performance, modeling technique, and feature selection by wrapper and filter methods. The usefulness of the approaches has been assessed using a multisensor system database created by Rodriguez Gamboa et al. [19] and gathering measurements of wine quality.

Interesting results concerning model performance have been obtained. We calculated model performance using the standard leave-one-group-out and group shuffle cross-validation methods. The obtained results led us to the conclusion that the latter method more reliably estimated model performance; thus, it was used in our current calculations. We believe such a comparison and indication, that the model accuracy estimated by the standard leave-one-group-out cross-validation results is sometimes too optimistic, can be interesting to other researchers, especially newcomers in the field of e-nose data analysis.

As an important part of the results, we present details of cross-validation, including standard deviation of the cross-validated model accuracy, which allows us to more deeply understand the expected performance of the models when new data are used. This also gives an important perspective when accuracy improvements are compared. Unfortunately, such results are rarely presented in other reports.

All sensor choice combinations were included in the computations, and all types of modeling features were employed. The results presented in Figure 4 indicate the optimal sensor matrix consists of only three sensors. A comparison of model accuracy obtained with various numbers of features, listed in the Appendix A, chosen by the recursive forward selection method can be seen in Figure 5. We can notice that the accuracy of the model built on only five features reached a value of 96.0%. Further increasing the number of features can still improve the model’s performance, and for 15 features it reached an accuracy of 97.1%. What is significant is the performance obtained on a reduced number of features, even as little as five, which was better than the best obtained model accuracy on the whole set of 828 features. Models trained on a smaller number of features will be more stable and less susceptible to overfitting. For such a reason, no more than 10 features would be the optimal choice. We also present the standard deviation of model accuracy obtained by the cross-validation procedure. Similarly, at this point, one can observe that the choice of more than 10 modeling features did not improve model stability. Therefore, a smaller number of features leads not only to better model performance in terms of accuracy, but it also improves the stability, as noted by the standard deviation of 5.6%. This means that we can expect a better performance of such a model on new, unseen data.

In our tests the model with the best choice of features had an accuracy of 98.4% with a cross-validation standard deviation of 3.4%. This result is better than the best one reported by Rodriguez Gamboa [19] (97.7%). One has to keep in mind that our estimates were obtained with a different cross-validation scheme, which, as we demonstrated, gave less optimistic estimates of classification accuracy. We believe that such a result may be encouraging to re-examine some publicly available datasets.

Looking at the data extracted from just one sensor, such as the 2nd, 3rd, or 5th sensors, we can see from Figure 6 that the model accuracy was about 95.5%, which is very close to the performance of models trained on features extracted from all sensors. We can also observe that for the models trained on data extracted from only the 2nd sensor, the standard deviation of accuracy, interpreted as a kind of model stability, was comparable to that obtained for the model trained on all data coming from all sensors. In our opinion, this result may be especially interesting from the potential applications perspective. The possibility of constructing e-noses for special purposes, based on single sensor, and to detect odors, which were not targeted by sensors producers, is sometimes overlooked.

Another purpose was to verify if there were any important differences between models trained on features extracted from resistance *R* or conductance *G* alone, or both. The second issue that we addressed was the possibility to use only part of the measurement characteristics, for example only the gas adsorption part of the measurement. One can observe that when data from all sensors were used, the model accuracy did not depend on the choice of features extracted from *G*, *R*, or both. The adsorption part of the characteristics appears to be sufficient, but in such a case to achieve a good model performance, it is required to use features extracted from sensor conductance. When we examine models trained on the data extracted from a single sensor response, the best performing models were trained on the whole adsorption and desorption parts of the response curve. These observations confirm the importance of the information contained in the desorption curve that can improve the odor recognition capability of e-noses.

Finally, one should mention the demonstrated advantage of wrapper selection over filter selection methods, visible in Figure 8. Even if such an observation has been already reported, we believe that detailed demonstrations of differences between the two methods can be interesting guidance for other research.

## Appendix A. List of aLl Features Used for Modeling

As we explained in Section 3.1 above, the first step in reducing the dimensions consists of extracting several features that describe the sensor response curve. Below we present a list of these features. In the feature names we used the following convention; the first character indicates if this feature is calculated based on resistance or conductance characteristics, and the second character indicates the sensor number (in this case, 1 to 6). Thus, a total of 828 features were extracted from the original sensor response curves.

**Basic statistics calculated from the whole response curve**	
R1.Sum/G1.Sum	Sum of sensor responses, which is equivalent to integral of the response curve.
R1.Median/G1.Median	Median
R1.Kurt/G1.Kurt	Kurtosis
R1.Skew/G1.Skew	Skewness

**Basic statistics calculated from the adsorption phase of the response curve.**	
R1.SumOn/G1.SumOn	Sum of sensor responses.
R1.MedOn/G1.MedOn	Median
R1.MinOn/G1.MaxOn	Extreme value reached by the response curve, which is equivalent to the value of the response at the end of the adsorption phase.

**Basic statistics calculated from the desorption phase of the response curve.**	
R1.SumOff/G1.SumOff	Sum of sensor responses.
R1.MedOff/G1.MedOff	Median
R1.MaxOff/G1.MinOff	Extreme value reached by the response curve, which is equivalent to the value of the response at the end of measurement.

**Time needed to reach the indicated percent change of the sensor response value during the adsorption phase (from baseline to extreme).**
R1.On10/G1.On10 10% R1.On25/G1.On25 25% R1.On50/G1.On50 50% R1.On75/G1.On75 75%

**Time needed to reach the indicated percent change of the sensor response value during the desorption phase (from start of desorption to end of the measurement).**
R1.Off10/G1.Off10 10% R1.Off25/G1.Off25 25% R1.Off50/G1.Off50 50% R1.Off75/G1.Off75 75%

**Extreme value of exponential moving average filter (emaα) for indicated values of the α parameter. Calculated for adsorption phase.**
R1.PMin1/G1.PMax1 α=0.1 R1.PMin2/G1.PMax2 α=0.01 R1.PMin3/G1.PMax3 α=0.001

**Time needed to reach extreme values of the exponential moving average filter (emaα) of the α parameter. Calculated for adsorption phase.**
R1.PTime1/G1.PTime1 α=0.1 R1.PTime2/G1.PTime2 α=0.01 R1.PTime3/G1.PTime3 α=0.1

**Basic statistics calculated for the exponential moving average filter (emaα) for indicated values of the α parameter. Calculated for the adsorption phase.**
R1.PStd1/G1.PStd1 Standard deviation, α=0.1 R1.PStd2/G1.PStd2 Standard deviation, α=0.01 R1.PStd3/G1.PSkew3 Standard deviation, α=0.001 R1.PSkew1/G1.PSkew1 Skewness, α=0.1 R1.PSkew2/G1.PSkew2 Skewness, α=0.01 R1.PSkew3/G1.PSkew3 Skewness, α=0.001 R1.PKurt1/G1.PKurt1 Kurtosis, α=0.1 R1.PKurt2/G1.PKurt2 Kurtosis, α=0.01 R1.PKurt3/G1.PKurt3 Kurtosis, α=0.001

**Extreme value of exponential moving average filter (emaα) for indicated values of the α parameter. Calculated for the desorption phase.**
R1.QMax1/G1.QMin1 α=0.1 R1.QMax2/G1.QMin2 α=0.01 R1.QMax3/G1.QMin3 α=0.001

**Time needed to reach the extreme value of the exponential moving average filter (emaα) for indicated values of the α parameter. Calculated for the desorption phase.**
R1.QTime1/G1.QTime1 α=0.1 R1.QTime2/G1.QTime2 α=0.01 R1.QTime3/G1.QTime3 α=0.001

**Basic statistics calculated for the exponential moving average filter (emaα) for indicated values of the α parameter. Calculated for the adsorption phase.**
R1.QStd1/G1.Qtd1 Standard deviation, α=0.1 R1.QStd2/G1.QStd2 Standard deviation, α=0.01 R1.QStd3/G3.QStd3 Standard deviation, α=0.001 R1.QSkew1/G1.QSkew1 Skewness, α=0.1 R1.QSkew2/G1.QSkew2 Skewness, α=0.01 R1.QSkew3/G1.QSkew3 Skewness, α=0.001 R1.QKurt1/G1.QKurt1 Kurtosis, α=0.1 R1.QKurt2/G1.QKurt2 Kurtosis, α=0.01 R1.QKurt3/G1.QKurt3 Kurtosis, α=0.001

**Value reached by the sensor response at time when the exponential moving average filter (emaα) reached its extreme. For indicated value of the α parameter.**
**Calculated for the desorption phase.**
R1.ValPMin1/G1.ValPMax1 α=0.1 R1.ValPMin2/G1.ValPMax2 α=0.01 R1.ValPMin3/G1.ValPMax3 α=0.001

**Parameters of sensor response curve fitting by polynomial function $y=A_{3}x^{3}+A_{2}x^{2}+A_{1}x+A_{0}$.**
R1.Poly3/G1.Poly3 $A_{3}$ R1.Poly2/G1.Poly2 $A_{2}$ R1.Poly1/G1.Poly1 $A_{1}$ R1.Poly0/G1.Poly0 $A_{0}$

**Values of the response curve at the 100×N-th sampling point. To avoid measurement noise, the median of ±5 points is taken.**
R1.v01 … R1.v15/G1.v01 .. G1.v15

## Appendix B. Features Selected by the Modeling Algorithm

In the table below, we present the best subset of features selected by the recursive forward selection method, for all types of subsets of features for which the results were described in the paper. The features are in order of selection by the algorithm. The first 10 features are listed.

**Data Range**	**Sensor**	**Features**
ALL	S1–S6	G6.Poly3, G4.PSkew3, R1.Std, G3.Poly2, G2.Kurt, G4.Poly3, G6.PStd1, G5.Kurt, G4.Poly2, R3.PSkew1
	S1	R1.Std, G1.QSkew1, R1.Off50, R1.QMax1, R1.PStd1, R1.ValPMin1, G1.QTime1, G1.Poly2, G1.QTime3, R1.On10
	S2	R2.PKurt1, G2.Off75, R2.PKurt3, G2.Kurt, G2.Off50, G2.QTime2, G2.Poly0, G2.Off25, R2.PSkew2, G2.On75,
	S3	G3.Off50, R3.Kurt, R3.PKurt2, R3.Off10, G3.PKurt3, G3.Poly3, G3.MinOff, G3.QTime3, G3.Off75, R3.PSkew1
	S4	G4.PSkew3, G4.Poly0, G4.QKurt3, G4.On10, G4.QStd1, G4.PSkew2, G4.QMmin1, G4.On25, G4.QSkew3, G4.PTime3
	S5	G5.Kurt, G5.ValPMax3, G5.PSkew1, G5.MinOff, G5.PKurt2, G5.QStd1, G5.Skew, G5.QKurt2, R5.Kurt, G5.On50
	S6	G6.Poly3, G6.QSkew1, G6.MinOff, G6.PSkew1, G6.QTime1, G6.PStd1, R6.PKurt3, G6.QKurt1, R6.ValPMin1, G6.PMax1

ALL-G	S1–S6	G6.Poly3, G4.PSkew3, G2.PTime3, G2.Kurt, G2.PKurt3, G5.PKurt2, G5.Off75, G4.PSkew2, G4.Poly3, G6.Kurt
	S1	G1.Poly0, G1.QKurt2, G1.QKurt3, G1.PSkew1, G1.QKurt1, G1.MinOff, G1.Off50, G1.QSkew1, G1.v02, G1.QSkew2
	S2	G2.PTime3, G2.Kurt, G2.PKurt2, G2.Skew, G2.QTime2, G2.QStd1, G2.MinOff, G2.On75, G2.PSkew2, G2.Off75
	S3	G3.Off25, G3.MinOff, G3.PStd1, G3.QSkew1, G3.PKurt2, G3.Poly2, G3.PKurt3, G3.Poly3, G3.PKurt1, G3.QKurt1
	S4	G4.PSkew3, G4.Poly0, G4.QKurt3, G4.On10, G4.QStd1, G4.PSkew2, G4.QMmin1, G4.On25, G4.QSkew3, G4.PTime3
	S5	G5.Kurt, G5.ValPMax3, G5.PSkew1, G5.MinOff, G5.PKurt2, G5.QStd1, G5.Skew, G5.QKurt2, G5.On50, G5.PSkew2
	S6	G6.Poly3, G6.QSkew1, G6.MinOff, G6.PSkew1, G6.QTime1, G6.PStd1, G6.QKurt1, G6.v04, G6.PSkew3, G6.PMax1

ALL-R	S1–S6	R1.Std, R5.Off25, R2.Kurt, R5.On10, R3.PMin1, R6.Off50, R6.PKurt3, R2.Skew, R6.Off10, R1.SumOff
	S1	R1.Std, R1.QMax1, R1.PTime1, R1.ValPMin1, R1.SumOff, R1.v05, R1.QTime1, R1.PStd1, R1.Kurt, R1.MedOff
	S2	R2.PKurt1, R2.Kurt, R2.PKurt3, R2.QMax3, R2.Off75, R2.QKurt3, R2.Skew, R2.PMin1, R2.On75, R2.PSkew2
	S3	R3.Kurt, R3.PKurt2, R3.Off10, R3.Std, R3.Skew, R3.QKurt2, R3.PKurt3, R3.PSkew2, R3.QKurt3, R3.QSkew2
	S4	R4.Std, R4.MinOn, R4.ValPMin3, R4.v08, R4.PStd2, R4.SumOff, R4.v02, R4.MedOn, R4.PStd1, R4.PStd3
	S5	R5.Std, R5.Skew, R5.PTime3, R5.Off10, R5.PMin1, R5.Off75, R5.Kurt, R5.On75, R5.MedOff, R5.SumOff
	S6	R6.Std, R6.On10, R6.SumOff, R6.On25, R6.Off25, R6.ValPMin1, R6.v01, R6.MedOff, R6.Off75, R6.Off50
ON	S1–S6	G6.Poly3, G4.PSkew3, G2.PTime3, G5.PKurt2, G3.PStd1, G4.v15, G2.PKurt2, R3.PMin1, G1.v01, G5.PSkew3
	S1	R1.PTime1, G1.PKurt1, R1.PStd1, R1.PTime3, R1.ValPMin2, R1.PTime2, G1.v02, R1.On10, R1.On25, G1.PKurt2
	S2	R2.PKurt1, G2.PSkew1, G2.PKurt3, G2.On75, R2.PKurt3, G2.PTime3, G2.PKurt2, G2.PSkew2, R2.ValPMin2, G2.PSkew3

	S3	G3.v01, G3.On50, G3.PSkew1, R3.PKurt3, G3.On75, G3.PKurt1, G3.Poly3, R3.On10, R3.PKurt1, G3.PKurt2
	S4	G4.PSkew3, G4.Poly0, G4.On75, G4.PTime3, G4.PKurt1, G4.PSkew2, G4.On25, G4.PKurt2, G4.On10, G4.On50
	S5	G5.On75, G5.ValPMax3, G5.PSkew1, G5.PSkew3, G5.ValPMax1, G5.Poly0, G5.Poly1, G5.PStd1, R5.ValPMin1, G5.v10
	S6	G6.Poly3, G6.PSkew1, G6.On25, G6.On50, G6.On75, G6.On10, G6.PTime1, G6.Poly2, G6.PTime3, R6.PTime1
ON-G	S1–S6	G6.Poly3, G4.PSkew3, G2.PTime3, G5.PKurt2, G3.PStd1, G4.v15, G2.PKurt2, G6.PMax1, G5.PSkew3, G1.v02
	S1	G1.Poly0, G1.On75, G1.PKurt1, G1.PKurt2, G1.Poly3, G1.On10, G1.ValPMax3, G1.v01, G1.PStd1, G1.On50
	S2	G2.PTime3, G2.PSkew1, G2.On75, G2.PKurt2, G2.ValPMax2, G2.On50, G2.PSkew2, G2.PTime2, G2.PKurt1, G2.PSkew3
	S3	G3.v01, G3.On50, G3.PSkew1, G3.Poly3, G3.On75, G3.PKurt1, G3.PTime1, G3.PTime3, G3.v02, G3.On25
	S4	G4.PSkew3, G4.Poly0, G4.On75, G4.PTime3, G4.PKurt1, G4.PSkew2, G4.On25, G4.PKurt2, G4.On10, G4.On50
	S5	G5.On75, G5.ValPMax3, G5.PSkew1, G5.PSkew3, G5.ValPMax1, G5.Poly0, G5.Poly1, G5.PStd1, G5.PStd2, G5.PStd3
	S6	G6.Poly3, G6.PSkew1, G6.On25, G6.On50, G6.On75, G6.On10, G6.PTime1, G6.Poly2, G6.PTime3, G6.PSkew3, G6.PStd1
ON-R	S1–S6	R2.PKurt1, R6.PTime1, R5.ValPMin3, R1.v08, R4.MinOn, R1.PTime1, R4.v03, R1.PMin2, R4.ValPMin1, R2.PSkew2
	S1	R1.PTime1, R1.PStd1, R1.ValPMin2, R1.PTime2, R1.MedOn, R1.On10, R1.PTime3, R1.PMin2, R1.v03, R1.v02
	S2	R2.PKurt1, R2.v01, R2.PKurt3, R2.PSkew3, R2.On75, R2.PSkew2, R2.PSkew1, R2.PKurt2, R2.PMin3, R2.PMin1
	S3	R3.v01, R3.PKurt3, R3.PKurt1, R3.v15, R3.On10, R3.PSkew2, R3.PTime2, R3.PMin1, R3.PSkew3, R3.PStd3
	S4	R4.PKurt1, R4.MedOn, R4.ValPMin3, R4.PSkew3, R4.v14, R4.v02, R4.PStd3, R4.PMin1, R4.PMin3, R4.v15
	S5	R5.ValPMin3, R5.On75, R5.ValPMin2, R5.v15, R5.On10, R5.MedOn, R5.PMin1, R5.v09, R5.v03, R5.v07
	S6	R6.PStd2, R6.PSkew2, R6.On10, R6.v07, R6.On75, R6.MinOn, R6.v01, R6.v06, R6.PSkew3, R6.v15

## References

[1] Hurot C., Scaramozzino N., Buhot A., Hou Y. (2020). Bio-Inspired Strategies for Improving the Selectivity and Sensitivity of Artificial Noses: A Review. Sensors.

[2] Wang D., Loo J., Chen J., Yam Y., Chen S.C., He H., Kong S., Ho H. (2019). Recent Advances in Surface Plasmon Resonance Imaging Sensors. Sensors.

[3] Brenet S., John-Herpin A., Gallat F.X., Musnier B., Buhot A., Herrier C., Rousselle T., Livache T., Hou Y. (2018). Highly-Selective Optoelectronic Nose Based on Surface Plasmon Resonance Imaging for Sensing Volatile Organic Compounds. Anal. Chem..

[4] Capelli L., Sironi S., Del Rosso R. (2014). Electronic Noses for Environmental Monitoring Applications. Sensors.

[5] Zhang D., Guo D., Yan K. (2017). Breath Analysis for Medical Applications.

[6] Berna A. (2010). Metal Oxide Sensors for Electronic Noses and Their Application to Food Analysis. Sensors.

[7] Baldwin E.A., Bai J., Plotto A., Dea S. (2011). Electronic Noses and Tongues: Applications for the Food and Pharmaceutical Industries. Sensors.

[8] Gliszczyńska-Świgło A., Chmielewski J. (2017). Electronic Nose as a Tool for Monitoring the Authenticity of Food. A Review. Food Anal. Methods.

[9] Rodríguez-Méndez M.L., De Saja J.A., González-Antón R., García-Hernández C., Medina-Plaza C., García-Cabezón C., Martín-Pedrosa F. (2016). Electronic Noses and Tongues in Wine Industry. Front. Bioeng. Biotechnol..

[10] Lozano J., Santos J.P., Aleixandre M., Sayago I., Gutierrez J., Horrillo M.C. (2006). Identification of typical wine aromas by means of an electronic nose. IEEE Sens. J..

[11] Lozano J., Santos J., Arroyo T., Aznar M., Cabellos J., Gil M., Horrillo M. (2007). Correlating e-nose responses to wine sensorial descriptors and gas chromatography–mass spectrometry profiles using partial least squares regression analysis. Sens. Actuators B Chem..

[12] Lozano J., Arroyo T., Santos J.P., Cabellos J., Horrillo M.C. (2008). Electronic nose for wine ageing detection. Sens. Actuators B Chem..

[13] Lozano J., Santos J.P., Horrillo M.C. (2008). Enrichment sampling methods for wine discrimination with gas sensors. J. Food Comp. Anal..

[14] Aguilera T., Lozano J., Paredes J.A., Álvarez F.J., Suárez J.I. (2012). Electronic Nose Based on Independent Component Analysis Combined with Partial Least Squares and Artificial Neural Networks for Wine Prediction. Sensors.

[15] Macías M., Manso A., Orellana C., Velasco H., Caballero R., Chamizo J. (2012). Acetic Acid Detection Threshold in Synthetic Wine Samples of a Portable Electronic Nose. Sensors.

[16] Rodriguez-Mendez M.L., Apetrei C., Gay M., Medina-Plaza C., de Saja J.A., Vidal S., Aagaard O., Ugliano M., Wirth J., Cheynier V. (2014). Evaluation of oxygen exposure levels and polyphenolic content of red wines using an electronic panel formed by an electronic nose and an electronic tongue. Food Chem..

[17] Wei Z., Xiao X., Wang J., Wang H. (2017). Identification of the Rice Wines with Different Marked Ages by Electronic Nose Coupled with Smartphone and Cloud Storage Platform. Sensors.

[18] Liu H., Li Q., Yan B., Zhang L., Gu Y. (2019). Bionic Electronic Nose Based on MOS Sensors Array and Machine Learning Algorithms Used for Wine Properties Detection. Sensors.

[19] Rodriguez Gamboa J.C., Albarracin E.E.S., da Silva A.J., de Andrade Lima L.L., Ferreira T.A.E. (2019). Wine quality rapid detection using a compact electronic nose system: Application focused on spoilage thresholds by acetic acid. Lwt-Food Sci. Technol..

[20] Rodriguez Gamboa J.C., Albarracin E.E.S., da Silva A.J., Ferreira T.A.E. (2019). Electronic nose dataset for detection of wine spoilage thresholds. Data Brief.

[21] Zhang L., Tian F., Zhang D. (2018). Book Review and Future Work. Electronic Nose: Algorithmic Challenges.

[22] Goodner K.L., Dreher J.G., Rouseff R.L. (2001). The dangers of creating false classifications due to noise in electronic nose and similar multivariate analyses. Sens. Actuators B Chem..

[23] Gardner J.W., Boilot P., Hines E.L. (2005). Enhancing electronic nose performance by sensor selection using a new integer-based genetic algorithm approach. Sens. Actuators B Chem..

[24] Phaisangittisagul E., Nagle H.T. (2008). Sensor Selection for Machine Olfaction Based on Transient Feature Extraction. IEEE Trans. Instrum. Meas..

[25] Phaisangittisagul E., Nagle H.T., Areekul V. (2010). Intelligent method for sensor subset selection for machine olfaction. Sens. Actuators B Chem..

[26] Guo D., Zhang D., Zhang L. (2011). An LDA based sensor selection approach used in breath analysis system. Sens. Actuators B Chem..

[27] Geng Z., Yang F., Wu N. (2011). Optimum design of sensor arrays via simulation-based multivariate calibration. Sens. Actuators B Chem..

[28] Zhang L., Tian F., Pei G. (2014). A novel sensor selection using pattern recognition in electronic nose. Measurement.

[29] Miao J., Zhang T., Wang Y., Li G. (2015). Optimal Sensor Selection for Classifying a Set of Ginsengs Using Metal-Oxide Sensors. Sensors.

[30] Sun H., Tian F., Liang Z., Sun T., Yu B., Yang S.X., He Q., Zhang L., Liu X. (2017). Sensor Array Optimization of Electronic Nose for Detection of Bacteria in Wound Infection. IEEE Trans. Ind. Electron..

[31] Tomic O., Eklöv T., Kvaal K., Huaugen J.E. (2004). Recalibration of a gas-sensor array system related to sensor replacement. Anal. Chim. Acta.

[32] Fonollosa J., Vergara A., Huerta R. (2013). Algorithmic mitigation of sensor failure: Is sensor replacement really necessary?. Sens. Actuators B Chem..

[33] Llobet E., Ionescu R., Al-Khalifa S., Brezmes J., Vilanova X., Correig X., Barsan N., Gardner J.W. (2001). Multicomponent gas mixture analysis using a single tin oxide sensor and dynamic pattern recognition. IEEE Sens. J..

[34] Szczurek A., Krawczyk B., Maciejewska M. (2013). VOCs classification based on the committee of classifiers coupled with single sensor signals. Chemometr Intell. Lab. Syst..

[35] Szczurek A., Maciejewska M. (2013). “Artificial sniffing” based on induced temporary disturbance of gas sensor response. Sens. Actuators B Chem..

[36] Hossein-Babaei F., Amini A. (2014). Recognition of complex odors with a single generic tin oxide gas sensor. Sens. Actuators B Chem..

[37] Herrero-Carrón F., Yáñez D.J., de Borja Rodríguez F., Varona P. (2015). An active, inverse temperature modulation strategy for single sensor odorant classification. Sens. Actuators B Chem..

[38] Pedregosa F., Varoquaux G., Gramfort A., Michel V., Thirion B., Grisel O., Blondel M., Prettenhofer P., Weiss R., Dubourg V. (2011). Scikit-learn: Machine Learning in Python. J. Mach. Learn Res..

[39] Brudzewski K., Ulaczyk J. (2009). An effective method for analysis of dynamic electronic nose responses. Sens. Actuators B Chem..

[40] Kaur R., Kumar R., Gulati A., Ghanshyam C., Kapur P., Bhondekar A.P. (2012). Enhancing electronic nose performance: A novel feature selection approach using dynamic social impact theory and moving window time slicing for classification of Kangra orthodox black tea (Camellia sinensis (L.) O. Kuntze). Sens. Actuators B Chem..

[41] Guo X., Peng C., Zhang S., Yan J., Duan S., Wang L., Jia P., Tian F. (2015). A Novel Feature Extraction Approach Using Window Function Capturing and QPSO-SVM for Enhancing Electronic Nose Performance. Sensors.

[42] Muezzinoglu M.K., Vergara A., Huerta R., Rulkov N., Rabinovich M.I., Selverston A., Abarbanel H.D.I. (2009). Acceleration of chemo-sensory information processing using transient features. Sens. Actuators B Chem..

[43] Vergara A., Vembu S., Ayhan T., Ryan M.A., Homer M.L., Huerta R. (2012). Chemical gas sensor drift compensation using classifier ensembles. Sens. Actuators B Chem..

[44] Eklöv T., Mårtensson P., Lundström I. (1997). Enhanced selectivity of MOSFET gas sensors by systematical analysis of transient parameters. Anal. Chim. Acta.

[45] Distante C., Leo M., Siciliano P., Persuad K.C. (2002). On the study of feature extraction methods for an electronic nose. Sens. Actuators B Chem..

[46] Zhang W., Liu T., Ye L., Ueland M., Forbes S.L., Su S.W. (2019). A novel data pre-processing method for odour detection and identification system. Sens. Actuators A Phys..

[47] Yan J., Guo X., Duan S., Jia P., Wang L., Peng C., Zhang S. (2015). Electronic Nose Feature Extraction Methods: A Review. Sensors.

[48] Marco S., Gutierrez-Galvez A. (2012). Signal and Data Processing for Machine Olfaction and Chemical Sensing: A Review. IEEE Sens. J..

[49] Li J., Cheng K., Wang S., Morstatter F., Trevino R.P., Tang J., Liu H. (2017). Feature selection: A data perspective. ACM Comput Surv..

[50] Cho J.H., Kurup P.U. (2011). Decision tree approach for classification and dimensionality reduction of electronic nose data. Sens. Actuators B Chem..

[51] Yan K., Zhang D. (2015). Feature selection and analysis on correlated gas sensor data with recursive feature elimination. Sens. Actuators B Chem..

[52] Gualdrón O., Brezmes J., Llobet E., Amari A., Vilanova X., Bouchikhi B., Correig X. (2007). Variable selection for support vector machine based multisensor systems. Sens. Actuators B Chem..

[53] Shi B., Zhao L., Zhi R., Xi X. (2013). Optimization of electronic nose sensor array by genetic algorithms in Xihu-Longjing Tea quality analysis. Math. Comput. Model..

[54] Wang X.R., Lizier J.T., Nowotny T., Berna A.Z., Prokopenko M., Trowell S.C. (2014). Feature Selection for Chemical Sensor Arrays Using Mutual Information. PLoS ONE.

[55] Wang X.R., Lizier J.T., Berna A.Z., Bravo F.G., Trowell S.C. (2015). Human breath-print identification by E-nose, using information-theoretic feature selection prior to classification. Sens. Actuators B Chem..

[56] Nowotny T., Berna A.Z., Binions R., Trowell S. (2013). Optimal feature selection for classifying a large set of chemicals using metal oxide sensors. Sens. Actuators B Chem..

[57] Yin Y., Chu B., Yu H., Xiao Y. (2014). A selection method for feature vectors of electronic nose signal based on Wilks Λ–statistic. J. Food Meas. Charact..

